# A Validity Analysis of Text-to-Image Generative Artificial Intelligence Models for Craniofacial Anatomy Illustration

**DOI:** 10.3390/jcm14072136

**Published:** 2025-03-21

**Authors:** Syed Ali Haider, Srinivasagam Prabha, Cesar A. Gomez-Cabello, Sahar Borna, Sophia M. Pressman, Ariana Genovese, Maissa Trabilsy, Andrea Galvao, Keith T. Aziz, Peter M. Murray, Yogesh Parte, Yunguo Yu, Cui Tao, Antonio Jorge Forte

**Affiliations:** 1 Division of Plastic Surgery, Mayo Clinic, Jacksonville, FL 32224, USA; 2 School of Dental, Unichristus, Fortaleza 60190-180, Brazil; 3 Department of Orthopedic Surgery, Mayo Clinic, Jacksonville, FL 32224, USA; 4 Center for Digital Health, Mayo Clinic, Rochester, MN 55905, USA; parte.yogesh@mayo.edu (Y.P.); yuyunguo@gmail.com (Y.Y.); 5 Department of AI and Informatics, Mayo Clinic, Jacksonville, FL 32224, USA

**Keywords:** artificial intelligence, generative AI, text-to-image, craniofacial anatomy, anatomy, generative adversarial networks, diffusion model

## Abstract

**Background:** Anatomically accurate illustrations are imperative in medical education, serving as crucial tools to facilitate comprehension of complex anatomical structures. While traditional illustration methods involving human artists remain the gold standard, the rapid advancement of Generative Artificial Intelligence (GAI) models presents a new opportunity to automate and accelerate this process. This study evaluated the potential of GAI models to produce craniofacial anatomy illustrations for educational purposes. **Methods:** Four GAI models, including Midjourney v6.0, DALL-E 3, Gemini Ultra 1.0, and Stable Diffusion 2.0 were used to generate 736 images across multiple views of surface anatomy, bones, muscles, blood vessels, and nerves of the cranium in both oil painting and realistic photograph styles. Four reviewers evaluated the images for anatomical detail, aesthetic quality, usability, and cost-effectiveness. Inter-rater reliability analysis assessed evaluation consistency. **Results:** Midjourney v6.0 scored highest for aesthetic quality and cost-effectiveness, and DALL-E 3 performed best for anatomical detail and usability. The inter-rater reliability analysis demonstrated a high level of agreement among reviewers (ICC = 0.858, 95% CI). However, all models showed significant flaws in depicting crucial anatomical details such as foramina, suture lines, muscular origins/insertions, and neurovascular structures. These limitations were further characterized by abstract depictions, mixing of layers, shadowing, abnormal muscle arrangements, and labeling errors. **Conclusions:** These findings highlight GAI’s potential for rapidly creating craniofacial anatomy illustrations but also its current limitations due to inadequate training data and incomplete understanding of complex anatomy. Refining these models through precise training data and expert feedback is vital. Ethical considerations, such as potential biases, copyright challenges, and the risks of propagating inaccurate information, must also be carefully navigated. Further refinement of GAI models and ethical safeguards are essential for safe use.

## 1. Introduction

### 1.1. Anatomical Illustrations: Cornerstones of Medical Education

“A picture is worth a thousand words,” as the adage goes, and this holds particularly true when it comes to the study of human anatomy. Anatomical illustrations are essential for understanding complex anatomy in medical education [1]. Traditional illustration methods involving skilled human artists remain the gold standard for creating detailed and precise anatomical representations [2]. These illustrations serve as crucial learning tools for medical professionals responsible for patient well-being and enhance the scientific community’s understanding of research papers [3].

### 1.2. Traditional Methods: Hurdles and Constraints

Becoming a medical illustrator requires significant time and specialized training, making their work highly valuable [4,5]. With fewer than 2000 trained medical illustrators worldwide [6], the global demand for medical education materials poses a significant challenge. Resource-constrained settings, especially in developing countries, face a difficult choice: invest heavily in creating original illustrations or purchase copyright permissions for existing ones. While AI-generated illustrations offer a potential solution, copyright concerns arise due to the models’ training on copyrighted datasets [7] Modern medical illustration employs a range of methods, from traditional hand drawing to sophisticated digital tools like ZBrush, Maya, Adobe Creative Suite, and other specialized medical visualization softwares [8]. Despite these advancements, challenges persist. Professional medical illustration demands specialized training and expertise, often requiring advanced degrees. Costs and time requirements vary significantly based on complexity, style, and intended use.

Previous research has shown that AI-generated medical illustrations can be both time- and cost-effective [3]. Traditional methods often require multiple 2D images to convey 3D anatomical structures, placing a cognitive burden on learners. While 3D models are increasingly available, they often lack customization and accessibility [9]. A more pressing issue is the lack of diversity in medical illustrations, with only 4.5% representing dark skin tones [10,11]. Recent studies have confirmed this bias, with a disproportionate representation of light skin tones (81.2%) and male bodies (61.6%) in medical textbooks [11]. This alarming statistic underscores the urgent need to address systemic biases in medical education and practice, highlighting the potential of AI-generated images to create more diverse and representative visual content.

### 1.3. GAI: A Promising Frontier

GAI advancements offer potential for automating and accelerating illustration. In 2022, text-to-image models, trained on vast datasets of images, emerged as a groundbreaking technology capable of generating novel, vivid, and imaginative visual content based on text prompts and descriptions. Researchers coined this technology “Autolography” [12]. Initially a research tool, these models have found various applications, including promising potential in medical illustration for creating anatomical images [13].

Large language models (LLMs) have demonstrated significant capabilities in accurately responding to queries about human anatomy [14,15,16]. The development of specialized GAI models, such as RadImageGan [17], which is specifically designed for generating diverse synthetic two-dimensional medical images, underscores the increasing focus on leveraging AI for medical image synthesis in research and training context. If GAI can achieve high levels of accuracy in creating and interpreting complex medical images for such crucial applications as disease diagnosis and scientific research, it logically follows that, with appropriate training and further refinement, it could also be capable of producing high-quality and anatomically accurate visual aids specifically designed for learning and instruction in the field of anatomy. Given the growing interest in AI-driven anatomy education and the promising results of LLMs, we sought to explore the potential of state-of-the-art text-to-image AI models in portraying craniofacial anatomy. Although prior work has explored the use of GAI in creating anatomical illustrations, there remains a gap in the literature regarding a systematic evaluation with a dedicated focus on craniofacial anatomy. In particular, the ability of these models to accurately and distinctly represent separate anatomical layers within the craniofacial region has not been thoroughly investigated [13,18].

This study aims to evaluate the potential of Midjourney v6.0, DALL-E 3, Gemini Ultra 1.0, and Stable Diffusion 2.0 for producing craniofacial anatomy images for educational purposes. By assessing factors like anatomical accuracy, aesthetic appeal, usability, and cost-effectiveness, we aim to determine their current state and potential applications in this domain.

## 2. Literature Review

### 2.1. Understanding AI Image Generation Techniques

**Figure 1:**
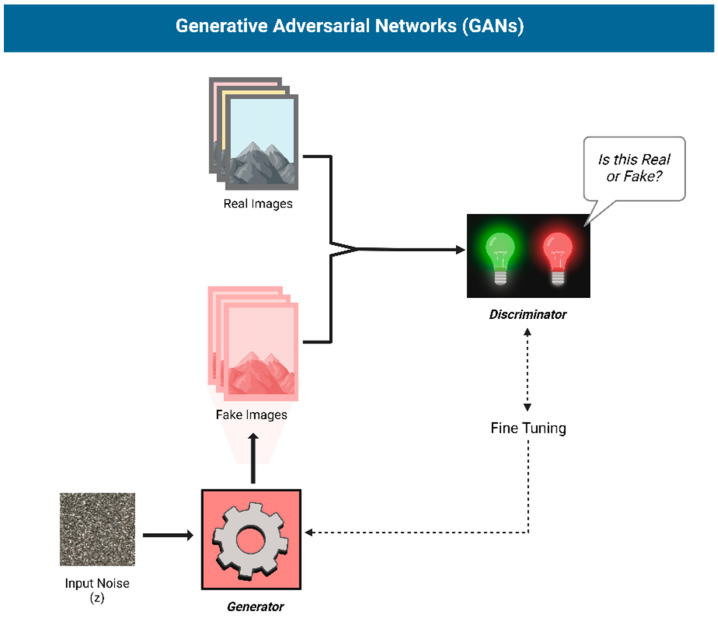
Structure of a Generative Adversarial Network (GAN): A Generator creates images, while a Discriminator distinguishes real from fake, refining both through adversarial training.

**Figure 2:**
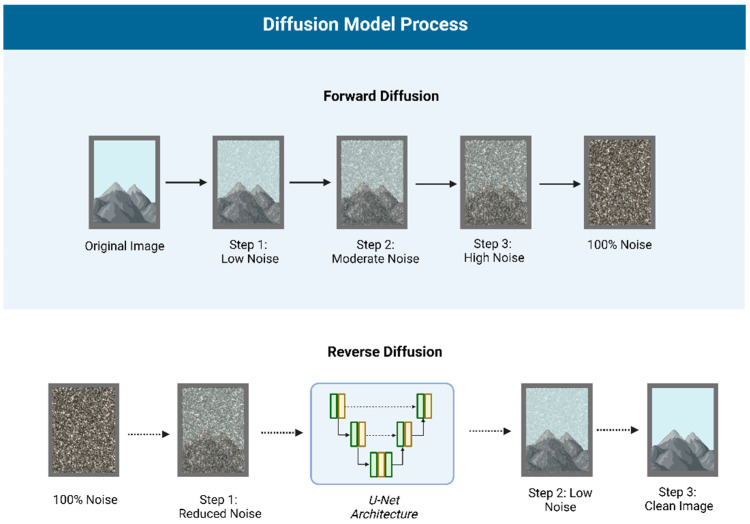
Diffusion Model Process: Forward diffusion gradually adds noise to an image, while reverse diffusion, using a U-Net architecture, reconstructs the original image by removing noise step by step.

AI text-to-image generation has evolved rapidly, with deep learning techniques like GANs or Generative Adversarial Networks (Figure 1) [19] and Diffusion Models (Figure 2) [20,21] powering modern models. GANs generate images through adversarial training, often producing sharp details but potential anatomical inaccuracies. Diffusion models, on the other hand, gradually add and remove noise, resulting in more stable, consistent outputs, albeit with potential loss of fine detail.

### 2.2. Applications in Medical Illustration

GAI models like Midjourney, DALL-E, and Stable Diffusion have gained significant attention for their potential in medical illustration [13,18,22,23,24,25,26]. These models offer advantages such as stylistic versatility, rapid prototyping, increased accessibility, efficiency, and aesthetically pleasing results [27]. Additionally, generated images are copyright-free [28].

Several studies have evaluated the capabilities and limitations of these models. Noel et al. investigated their performance in generating detailed illustrations of the human skull, heart, and brain [13]. Adams et al. examined DALL-E 2’s ability to generate and manipulate medical images [26]. Ajmera et al. validated DALL-E 3’s capacity to generate accurate illustrations of the musculoskeletal system [18]. Other studies have explored AI’s potential in creating images depicting clinical features, laboratory images, and various medical diagnostic modalities [22,23,24,25,26,29].

While these studies highlight the potential of AI, they also reveal limitations. Models often struggle with pathological findings and cross-sectional imaging modalities. Many AI-generated illustrations tend to be more artistic than strictly anatomically accurate [13,23].

## 3. Materials and Methods

### 3.1. GAI Models Evaluated

Four publicly available GAI models were selected for the creation and evaluation of craniofacial anatomy illustrations:

1. Midjourney (Paid v6.0, Midjourney, Inc., San Francisco, CA, USA) [28];
2. DALL-E (Paid, Model: 3, OpenAI, San Francisco, CA, USA) [30];
3. Gemini (Paid, Model: Ultra 1.0, Google, Mountain View, CA, USA) [31];
4. Stable Diffusion (Open-source, v2.0, Stability AI < CompVis < RunwayML) [32].

These models were chosen because they represent a range of leading GAI technologies, widely recognized and adopted in the field, and several are the most popular by market size. Midjourney and DALL-E are recognized for their high-quality image generation and artistic versatility, while Stable Diffusion is a widely used open-source model known for its flexibility and accessibility. Gemini Ultra 1.0 was included to evaluate one of the newer models by Google, offering a comparative perspective on the latest advancements in the field.

### 3.2. Image Generation Process

#### 3.2.1. Prompt Generation

We aimed to generate images in two styles—oil painting resembling a medical illustration and a professional photograph—as these formats are predominant in published anatomical literature. To achieve this, we generated images across five key anatomical layers: surface anatomy, bones, muscles, blood vessels, and nerves. For each layer, images were generated from six different views—frontal, lateral, superior, inferior, and mid-sagittal (n = 6 views × 5 layers = 30). Considering both the styles (30 × 2 = 60), this resulted in 60 prompts. However, due to the limited anatomical relevance of the mid-sagittal view for craniofacial muscles, these two prompts were excluded, resulting in a total of 58 unique prompts. Each prompt was repeated four times for the four AI models, initially aiming for a dataset of 928 images (58 × 4 × 4). However, due to errors with the Gemini Ultra 1.0 model in generating human faces, the final dataset consisted of 736 images.

A single basic version of the prompt was made, incorporating the different variations:

*“A high-definition [illustration/photograph] of the [layer: bone/muscle etc.] of the human [face/head], [frontal/lateral/superior/inferior/mid-sagittal] view, with a clear depiction of [features]. The background is simple and white. Style: [Oil painting in a medical illustration/ Photograph taken from a professional camera]”*

#### 3.2.2. Ethical Approval

The study utilized publicly available GAI technologies to create images using their own databases, so no patient information was used. Therefore, Institutional Review Board (IRB) permission was unnecessary.

### 3.3. Evaluation Criteria

The generated images were evaluated using a 5-point Likert scale across four key criteria, adapted from Ajmera et al. [18]. These criteria are consistent with evaluation metrics used in previous studies assessing the quality of medical illustrations and AI-generated images. We opted not to evaluate AI-generated annotations in the images, aligning with current research suggesting their general unreliability [18]. This approach mirrored our own experience with these models.

1. **Anatomical detail (D):** This criterion assessed the accuracy and completeness of the anatomical features depicted, including proportions, layers, angles, and views. This included accurate proportions of anatomical structures, clear delineation of the requested anatomical layer (e.g., nerves being distinguishable from surrounding structures, muscles having accurate origins/insertions), and appropriate viewing angles matching the specified prompts. Reviewers were asked to specifically assess the correctness of anatomical relationships and the presence of key anatomical landmarks.
2. **Aesthetic quality (A):** This criterion evaluated the visual appeal of the images, considering factors such as color, lighting, and shadows, in comparison to currently available publications while adhering to the requested artistic style specified in the prompt. Reviewers were asked to consider if the color palette and lighting were appropriate for medical illustrations, and if shadows enhanced or detracted from anatomical clarity
3. **Usability (U):** This criterion assessed the suitability of the images for use in scientific literature compared to currently available resources (i.e., is the current image usable in research papers, publications, books, or other educational materials). Reviewers were instructed to consider if the images could be used without further modification in a textbook or research article.
4. **Cost-effectiveness (C):** This criterion evaluated the potential cost savings of using AI-generated images compared to traditional illustration methods, based on the estimated number of potential revisions and the time and effort a professional artist would need to refine the AI-generated image to meet traditional standards. Reviewers were instructed to estimate the number of potential revisions and the time and effort required for a professional artist to achieve traditional standards.

### 3.4. Evaluation Process

Four reviewers, including medical professionals holding MD/MBBS degrees with anatomical expertise, independently evaluated the images. Each criterion was scored while keeping in reference the highly anatomically accurate illustrations from Netter and Sobotta’s anatomical atlases [32,33]. Images were randomized and presented blindly to minimize bias. Inter-rater reliability was assessed using a two-way mixed-effects model, and the Intraclass Correlation Coefficient (ICC) was calculated. The average measures ICC and its 95% confidence interval was reported to indicate the reliability of the average ratings. The analysis includes Average Measures ICC for overall agreement, agreement by model (Midjourney v6.0, DALL-E 3, etc.), and agreement by metric (anatomical detail, aesthetic quality, etc.). Additionally, the time taken by each AI model to generate images was recorded.

### 3.5. Craniofacial Proportion Index Calculation

**Figure 3:**
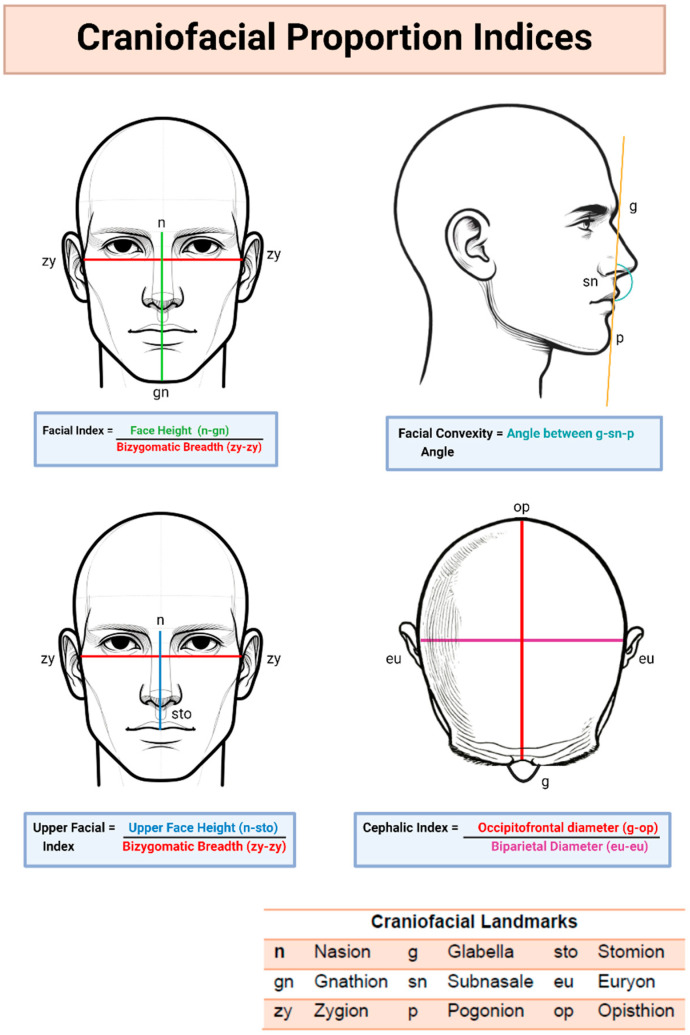
Craniofacial Proportion Index Calculation.

To assess the anatomical accuracy of the generated images, craniofacial proportion analysis was conducted using ImageJ software (v 1.54). Two independent reviewers measured specific landmarks on each image, following established photographic anthropometric analysis protocols [33,34,35,36,37]. The average measurements were compared to reference values from the Sobotta Atlas of Human Anatomy (Figure 3).

#### 3.5.1. Frontal View Analysis

- Facial Index: calculated from facial height and bizygomatic breadth, providing insights into facial proportions [37].
- Upper Facial Index: calculated from upper face height and bizygomatic breadth, providing insights into vertical facial proportions.

#### 3.5.2. Lateral View Analysis

- Facial Convexity Angle: measured to assess facial profile balance and protrusion [38].

#### 3.5.3. Superior View Analysis

- Cephalic Index: calculated from biparietal and occipitofrontal distances, providing insights into head shape [39].

## 4. Results

**Figure 4:**
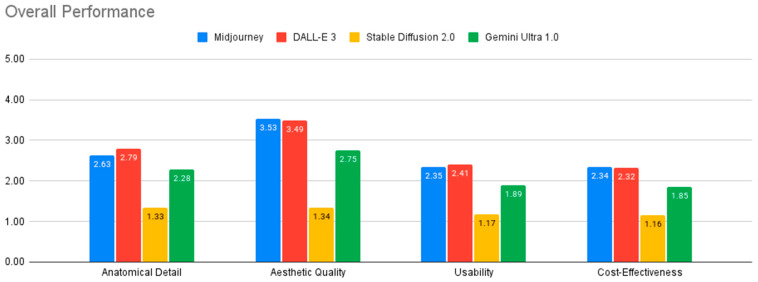
Overall performance of AI models.

Table 1 and Figure 4 summarize the performance evaluation of the four AI models across various criteria. All models fell short of the standards required for medical illustration, except in generating surface anatomy illustrations.

Among the models evaluated, DALL-E 3 and Midjourney v6.0 generally demonstrated the strongest performance, while Stable Diffusion 2.0 consistently showed the weakest results across all evaluation criteria. The analysis of variance (ANOVA) revealed statistically significant differences between the four AI models in their performance across all four evaluation criteria: anatomical detail, aesthetic quality, usability, and cost-effectiveness (all *p* < 0.001). Effect sizes, as measured by Eta-squared, ranged from medium to large, with the largest effect observed for aesthetic quality (Eta-squared = 0.387), indicating that model differences had a substantial impact on this criterion. Post-hoc analysis using Tukey’s HSD test further elucidated the nature of these differences.

In terms of anatomical detail, DALL-E 3 outperformed both Gemini Ultra and Stable Diffusion 2.0 (*p* < 0.001). Midjourney v6.0 also demonstrated significantly better anatomical detail than Stable Diffusion 2.0 (*p* < 0.001). Regarding aesthetic quality, DALL-E 3 and Midjourney v6.0 performed comparably (*p* = 0.867) and both significantly outperformed Gemini Ultra and Stable Diffusion 2.0 (*p* < 0.001). Stable Diffusion 2.0 received the lowest ratings for aesthetic quality. For usability, DALL-E 3 showed the highest performance, with significantly greater usability than Gemini Ultra and Stable Diffusion 2.0 (*p* < 0.001). Midjourney v6.0 also outperformed Stable Diffusion 2.0 in usability (*p* < 0.001).

### 4.1. Subgroup Analysis of AI Model Performance Across Anatomical Layers

**Figure 5:**
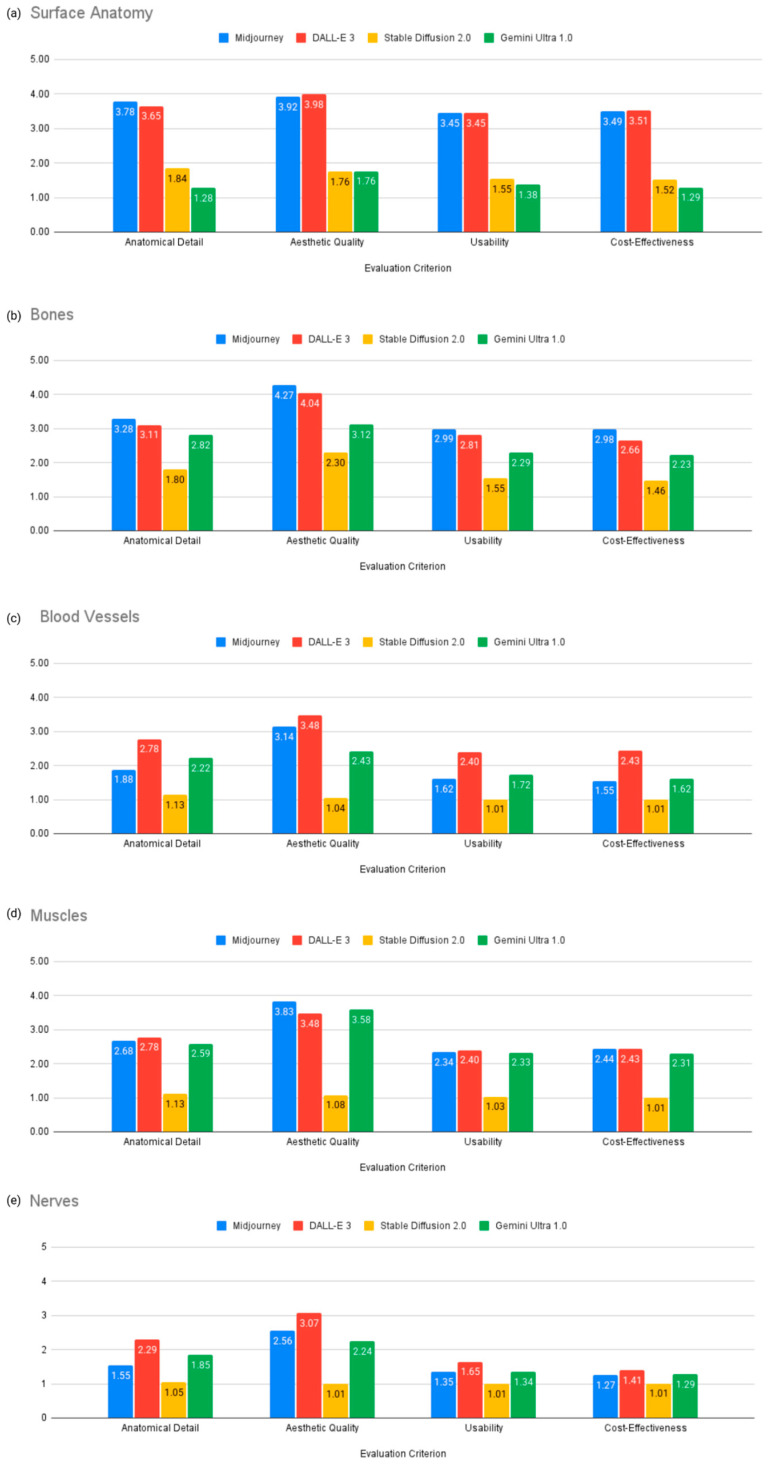
Subgroup analysis of AI model performance across anatomical layers.

Table 2 and Figure 5 present subgroup analysis results for each model’s performance in generating illustrations of specific anatomical layers (surface anatomy, bones, muscles, blood vessels, nerves).

- Surface Anatomy: Midjourney v6.0 performed best in anatomical detail with a score of 3.78, while DALL-E 3 was strongest in aesthetic quality with a score of 3.98 and cost-effectiveness with a score of 3.51 (Figure 5a).
- Bones: Midjourney v6.0 outperformed other models in all criteria, with a score of 3.28 for anatomical detail, 4.27 for aesthetic quality, 2.99 for usability, and 2.98 for cost-effectiveness (Figure 5b).
- Blood Vessels and Nerves: DALL-E 3 outperformed other models, including anatomical detail (2.78), aesthetic quality (3.48), usability (2.4), and cost-effectiveness (2.43) but the generated images were still not suitable for educational purposes due to anatomical inaccuracies (Figure 5c,e).
- Muscles: DALL-E 3 was better in anatomical detail with a score of 2.78 and usability with a score of 2.40, while Midjourney v6.0 was stronger in aesthetic quality with a score of 3.83 (Figure 5d).

**Figure 6:**
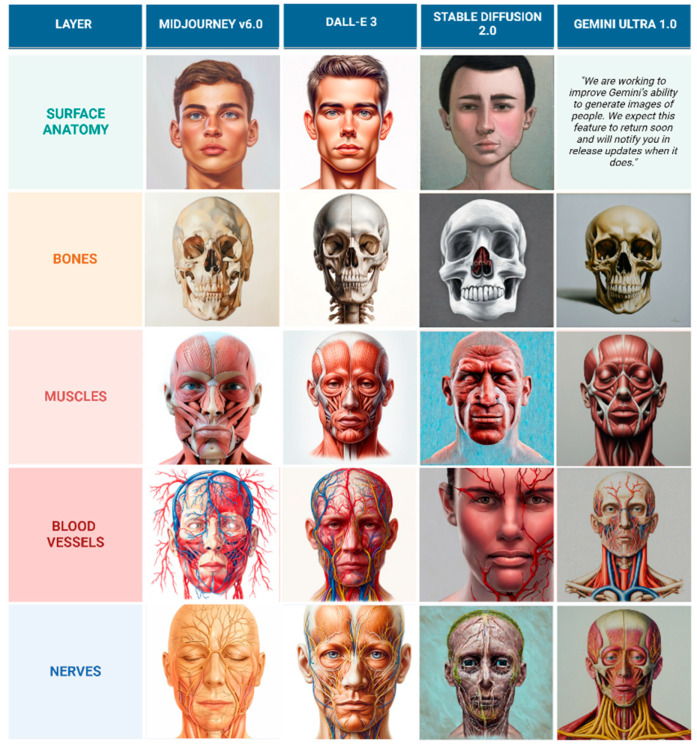
Comparison of AI-generated frontal view anatomical layers in oil painting style.

**Figure 7:**
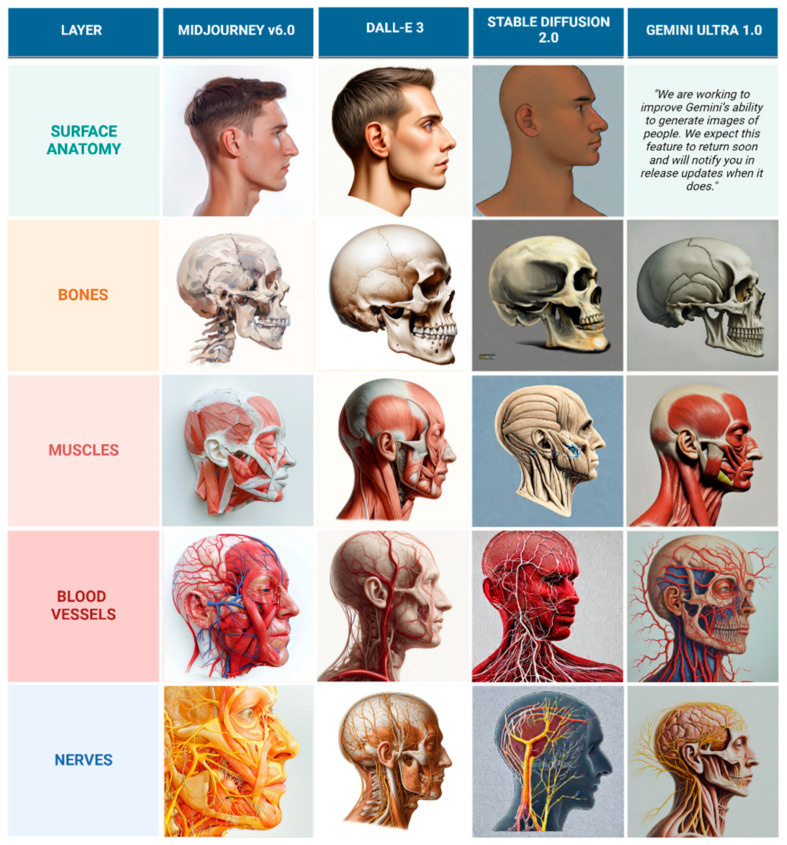
Comparison of AI-generated lateral view anatomical layers in oil painting style.

Despite DALL-E 3’s superior performance compared to other models, the generated illustrations are still not considered usable for educational purposes because the depicted nerves and vessels do not follow anatomically accurate courses. Sample of illustrations representing frontal view and lateral view are represented in Figure 6 and Figure 7.

### 4.2. Inter-Rater Reliability Analysis

The inter-rater reliability analysis revealed a high degree of overall agreement among reviewers (ICC = 0.858, 95% CI). The agreement was also high while assessing individual AI models and metrics. The results of the inter-rater reliability analysis are provided in Table 3:

### 4.3. Results of Cephalometric Analysis

The mean cephalometric measurements from the generated images were compared to reference values. DALL-E 3 aligned closest with reference values for Upper Facial Index (47.77 vs. 48.40), Facial Convexity Angle (171.00 vs. 170.96), and Cephalic Index (80.86 vs. 80.00). Midjourney v6.0 matched the reference Facial Index (86.63 vs. 86.22). Stable Diffusion 2.0 showed greater differences across all measurements, and Gemini Ultra 1.0’s analysis was limited to the Cephalic Index. The complete results are presented in Table 4.

## 5. Discussion

### 5.1. Performance Evaluation and Comparative Analysis

**Figure 8:**
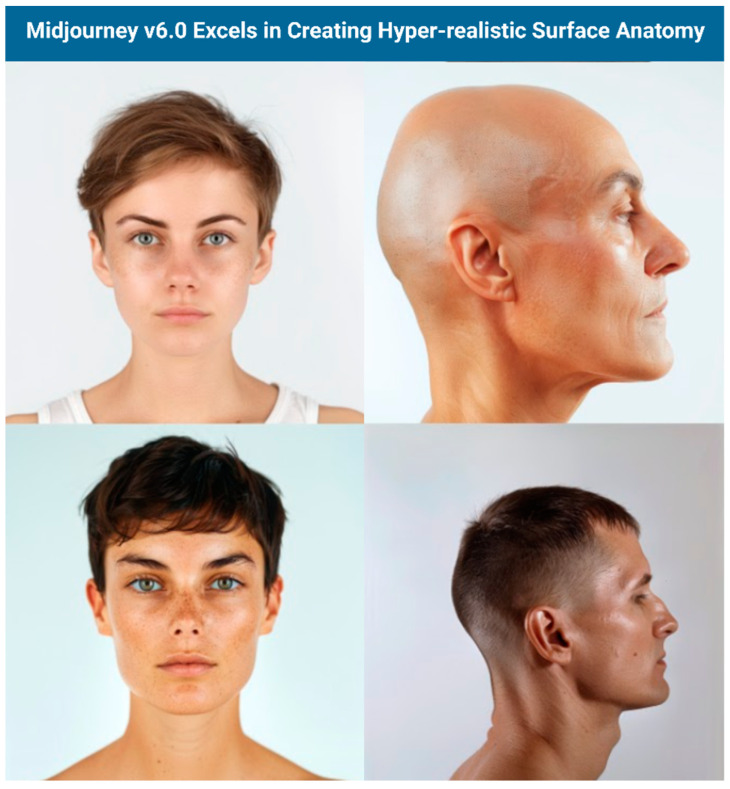
Surface Anatomy results from Midjourney.

Quantitative analysis revealed mixed performance across the evaluated models. DALL-E 3 led in anatomical detail (2.79 ± 1.15) and usability, while Midjourney v6.0 performed best in aesthetic quality (3.53 ± 1.3) and cost-effectiveness. Importantly, the analysis of variance (ANOVA) and Post-hoc Tukey test demonstrated statistically significant differences between the four AI models in their performance across all four evaluation criteria: anatomical detail, aesthetic quality, usability, and cost-effectiveness (all *p* < 0.005). Both models achieved sub-optimal scores for educational use, with particular difficulties in depicting crucial anatomical features like foramina, suture lines, and neurovascular structures. Midjourney V6.0 produced hyperrealistic, visually pleasing, and increasingly anatomically detailed images that can be useful for visualizing surface anatomy, though users should always verify accuracy with established medical resources (Figure 8).

Gemini’s strict content filters prevented generation of human surface anatomy [40], though it performed well with superior, inferior, and midsagittal views. Stable Diffusion 2.0 significantly underperformed, often producing histology-like images instead of clear anatomical structures.

All models generated images rapidly (<1 min) but required substantial refinement to approach medical education standards. Midjourney v6.0’s strength in aesthetics stemmed from its artistic training focus, while DALL-E 3’s broader training data enabled better handling of complex anatomical structures.

The high degree of agreement demonstrated in the inter-rater reliability analysis (ICC = 0.858, 95% Confidence Interval) indicates consistency among reviewers in assessing AI-generated illustrations. This consistency supports the validity of the study’s evaluation criteria and methodology, suggesting that the criteria were clear and well-defined. The reliability of the ratings strengthens the generalizability of the findings, increasing confidence that similar results could be obtained with a different group of reviewers. Furthermore, the high ICC reduces the potential impact of individual reviewer biases, demonstrating a relatively objective evaluation process. Nevertheless, the challenge of achieving consistent reproduction of sequential illustrations complicates the assessment of these models’ practical utility.

### 5.2. The Role of AI in Anatomy Education

Recent research has highlighted the potential applications of AI in the field of anatomy education and learning. Several studies have investigated the capabilities of language models in comprehending and explaining complex anatomical concepts and structures. In a series of examinations, ChatGPT has demonstrated a strong understanding of anatomical concepts and structures, consistently outperforming medical students [15]. AI has been shown to enhance medical education by assessing and reinforcing anatomical knowledge and generating personalized questions, clinical scenarios, and simulations tailored to individual learners. Moreover, research has been conducted to assess ChatGPT’s ability to answer questions and solve clinical scenarios related to head and neck surgery. Notably, it has also been evaluated for its adherence to clinical guidelines in craniofacial plastic and reconstructive surgeries, such as rhinoplasty and eyelid surgery, demonstrating a high degree of adherence to established best practices and providing valuable insights and recommendations [16,41,42].

The rapid advancement of GAI models presents a new opportunity to complement traditional methods in medical education, offering unique benefits in specific scenarios. For instance, GAI models can quickly generate visualizations of complex 3D relationships between anatomical structures, which can be particularly helpful for introductory anatomy or review purposes. It offers the flexibility to request multiple perspectives of the same anatomical structure, such as anterior, posterior, or lateral views, as well as the ability to emphasize specific features like nerve pathways within a muscle or the intricate branching patterns of blood vessels. This adaptability stands in stark contrast to the static nature of traditional illustrations found in textbooks, where the viewpoint and features depicted are fixed. GAI, therefore, provides a more flexible and adaptable approach to visualizing anatomy, allowing educators to generate visuals that directly address the learning objectives of a particular lesson or module.

Beyond static images, GAI-generated illustrations hold significant potential for integration into interactive learning platforms, virtual reality (VR) and augmented reality (AR) environments, and various other digital educational tools [43,44]. GAI could also be used to generate visuals that actively respond to student interactions within a learning platform, such as providing different layers of anatomical detail as a student hover over or clicks on a particular structure.

The integration of GAI into medical education offers a multitude of potential advantages, particularly in the realm of anatomical illustration. One significant benefit is the potential for increased student engagement. Dynamic and interactive visuals generated by GAI can capture student interest and make the learning process more captivating compared to static images in textbooks. Another key advantage is the potential for the efficient creation of diverse visual content by educators. Educators can quickly and easily generate a wide array of illustrations for lectures, presentations, and learning materials without needing extensive artistic skills or investing significant time.

GAI has the potential to offer stylistic versatility, rapid prototyping, increased accessibility, efficiency, and aesthetically pleasing illustrations. In resource-constrained environments, especially in developing countries, GAI can provide a cost-effective solution to the challenge of creating or acquiring anatomical illustrations. AI-generated images and videos can also be used for personalized learning materials and aiding in clinical decision-making.

### 5.3. Technical and Methodological Challenges

**Figure 9:**
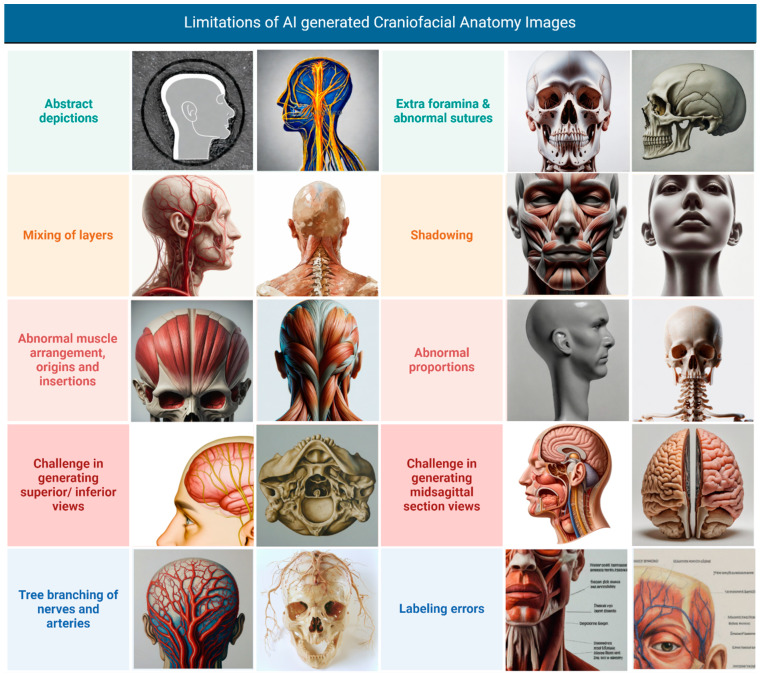
Limitations of AI-generated craniofacial anatomy illustrations. Collage created with Biorender.com ©.

Our study implemented iterative prompt refinement to minimize inaccuracies, developing detailed specifications for anatomical layers, views, and expected features. Despite these efforts, the models consistently exhibited significant limitations in accurately depicting crucial anatomical features, suggesting insufficient training on specialized anatomical datasets. As illustrated in Figure 9, these limitations included the following:

- Abstract Depictions: some images presented overly simplified or stylized representations of anatomical structures, lacking the necessary detail for medical education;
- Mixing of Layers: the models often juxtaposed anatomical layers, such as including flesh and skin on the nose in lateral views of the skull bones or depicting superficial muscles alongside deep structures;
- Abnormal Muscle Arrangement, Origins, and Insertions: muscle anatomy was frequently inaccurate, with errors in muscle shape, arrangement, and the depiction of origins and insertions;
- Challenge in Generating Superior/Inferior Views: the models struggled to accurately generate superior and inferior views, often resulting in distorted or incomplete representations;
- Tree Branching of Nerves and Arteries: the depiction of blood vessels and nerves often lacked a clear course and distribution, sometimes appearing to emerge from the body and project into the surrounding area like tree branches, rather than following realistic anatomical pathways;
- Extra Foramina and Abnormal Sutures: skeletal representations often included extra foramina or depicted abnormal suture lines, deviating from accurate anatomical structure;
- Shadowing: in some instances, shadowing was unrealistic or obscured anatomical details, hindering clarity;
- Abnormal Proportions: images from Stable Diffusion 2.0 exhibited abnormal proportions in the depicted anatomical structures;
- Challenge in Generating Midsagittal Section Views: the models encountered difficulties in generating accurate midsagittal section views, with structures often appearing incomplete or misrepresented;
- Labeling Errors: when present, annotations and labels were frequently illegible or nonsensical in line with previous research [45].

Evaluators also noted that the models often failed to accurately render specific foramina, such as the foramen ovale, and struggled to depict muscular attachments accurately, with frequent inaccuracies in the temporalis muscle’s insertion on the coronoid process. The path of the facial nerve was inconsistent and often included extraneous branches. Skulls often had inaccurate suture lines, depicted as continuous lines rather than serrated edges, and tooth counts frequently deviated from the standard 32 teeth. Furthermore, the models exhibited a tendency to ’hallucinate’ non-existent bones or fuse existing ones, with frequent inaccuracies observed in the depiction of the cervical vertebrae.

These results suggest the text-to-image AI models are currently insufficiently trained on specialized anatomical datasets [46]. A significant concern is the models’ bias toward conventionally attractive, thin individuals with lighter skin tones. This observation aligns with current literature highlighting how AI image generators can perpetuate demographic stereotypes [47]. The predominance of male figures and limited diversity in ethnicity and body types reflect inherent biases in the training data.

### 5.4. Ethical Considerations

The use of GAI in medical illustration presents ethical challenges that require careful attention. Bias in training data or models can perpetuate stereotypes and inaccuracies, necessitating robust evaluation and debiasing mechanisms [48,49]. Copyright and intellectual property concerns arise when AI generates illustrations based on existing medical imagery or text. The opacity of AI training data complicates attribution and compliance with Creative Commons licenses, highlighting the need for clear legal frameworks to protect creators’ rights [50].

Research has quantitatively demonstrated the underrepresentation of darker skin tones and women in AI-generated medical and professional imagery [51]. This bias stems from various sources throughout the AI lifecycle, including skewed training data that often lacks diversity, algorithmic design that can inadvertently rely on demographic shortcuts, and human biases present in data labeling and model development [52].

Beyond these immediate concerns, there are long-term ethical implications to consider. GAI models have the potential to disrupt established practices in medical illustration, potentially impacting the role of human medical illustrators and requiring a shift in the skills and expertise required for this field. This disruption could affect the medical illustration industry as a whole, with implications for various stakeholders. Medical illustrators may face job displacement or need to adapt their skills, while medical educators and publishers might experience cost savings and faster production times but also face potential quality concerns. Patients could benefit from improved educational materials but also face risks associated with inaccurate illustrations. The potential economic impact of GAI on the medical illustration market, including pricing pressures and new business models, also warrants consideration. There is a need to establish new professional standards and ethical guidelines for the use of GAI in medical illustration, with professional organizations playing a crucial role in guiding the responsible adoption of this technology. AI-generated illustrations also pose a potential threat to the creative expression of human medical illustrators, with concerns about AI mimicking artists’ styles without proper compensation—paralleling debates from the rise of photography and digital art. Mechanisms to ensure fair remuneration for illustrators whose work informs AI models are essential [53]. Lastly, the risk of propagating inaccurate or misleading anatomical information demands stringent quality control measures, including expert review and validation, to uphold the accuracy and integrity of medical education.

### 5.5. Future Directions

Advancing the quality of AI-generated anatomical illustrations requires robust collaboration between AI researchers, medical illustrators, and anatomists to develop specialized training datasets and assessment frameworks. Given the novelty and rapid evolution of AI-generated medical illustrations, there is an urgent need for the development of appropriate evaluation scales and verification criteria. Additional training with specialized image datasets encompassing a wider range of angles is crucial to further enhance the models’ capabilities. Specialized datasets could encompass collections of medical images (e.g., CT scans, MRI scans) with expert annotations, datasets of detailed anatomical illustrations from textbooks and atlases, or 3D anatomical models. Models like CephGPT-4 have demonstrated the potential for accurate analysis of anatomical proportions [54], suggesting that specialized datasets can facilitate precise anatomical representations. Emerging technologies, including Google’s Imagen 3 for improved text rendering [55] and text-to-3D models like NVIDIA’s Magic 3D, Dreamfusion [56], and Alpha3D [57], could overcome 2D representation constraints. The recent launch of OpenAI’s Sora AI for text-to-video conversion opens new possibilities for dynamic anatomical visualization [58].

A promising area to explore is the adaptation of these models in real-time based on expert feedback. This could involve iterative training processes where user feedback is directly integrated into the model to refine its output. For example, a user interface within the image generation tool could allow medical professionals to highlight inaccuracies and provide specific corrections, with AI-assisted analysis of this feedback to automatically adjust model parameters. Prompt engineering, the process of crafting effective text prompts to guide AI image generation, can also be leveraged to improve accuracy and specificity in real-time. Techniques such as using specific anatomical terminology, specifying desired views and details, and providing negative prompts can be employed. User feedback can be invaluable in refining prompt engineering efforts, leading to more effective prompts over time. Fine-tuning, which involves further training a pre-existing AI model on a specialized dataset, can also tailor AI models to the specific requirements of medical illustration. User feedback can guide the fine-tuning process, ensuring that the model learns to generate images that meet the needs of medical professionals.

Incorporating user feedback from medical professionals who would use these images in practice is crucial to add an important layer of real-world context and ensure their practical utility. Methods for gathering user feedback could include surveys, focus groups, interviews, and pilot studies where medical professionals use the AI-generated images in real-world settings (e.g., teaching, patient education) and provide feedback on their experience. This feedback can then inform further model development and refinement, ensuring that the generated images meet the specific needs and requirements of their end-users.

To further solidify this path and unlock the full potential of this technology, several promising avenues merit exploration. First, Human-in-the-Loop Integration could be developed, where human guidance could be employed for prompt creation, image refinement, or anomaly detection within the generated illustrations, ensuring the accuracy and realism of the output [59,60]. Second, Data augmentation techniques, such as flipping, rotating, scaling, and elastic deformation of existing training images, can be used to enhance the training data and improve the LLMs ability to generate a wider variety of anatomical structures and views, addressing the challenges we observed in generating accurate superior/inferior and midsagittal views [61].

Retrieval-augmented generation (RAG) is a promising approach to enhancing the performance of text-to-image AI models by incorporating external knowledge sources, such as anatomical databases and expert knowledge, into the image-generation process to improve the accuracy of anatomical depictions and relationships between structures [62]. Traditional text-to-image models rely solely on their internal knowledge, which may be incomplete or outdated. RAG addresses this limitation by retrieving relevant information from external sources, such as databases, websites, or pre-trained models, to augment the input text prompt and guide the image generation process.

Another avenue worth researching is the influence of demographic-specific prompts on the generated results. Incorporating age, gender, and race-specific information into the prompts could potentially yield more diverse and representative anatomical illustrations. Finally, Adversarial training methods could be implemented, where a separate discriminator model is trained to specifically identify and penalize anatomical inaccuracies (e.g., incorrect muscle attachments, inaccurate bone structures) within the LLM’s generated images. Through this adversarial process, the LLM’s ability to produce highly accurate illustrations would be iteratively refined as the generator and discriminator models continuously challenge and improve each other [63].

## 6. Conclusions

Our systematic evaluation of AI models for craniofacial anatomical illustrations revealed significant limitations in current capabilities. While Midjourney v6.0 and DALL-E 3 performed better than competitors (*p* < 0.05), neither met acceptable medical education standards. Midjourney v6.0 performed well in aesthetics but lacked anatomical accuracy, while DALL-E 3 showed marginally better anatomical detail but remained inadequate for educational use.

Despite rapid generation speeds and Midjourney v6.0’s cost-effectiveness, the extensive corrections needed to achieve medical accuracy would nullify these advantages. Our craniofacial proportion analysis confirmed substantial deviations from established anatomical references across all models.

Future development must balance AI’s speed and artistic capabilities with the strict requirements for anatomical accuracy and educational utility. Success requires interdisciplinary collaboration between AI researchers, medical illustrators, and anatomists, along with rigorous validation protocols. Until these improvements are achieved, traditional illustration methods remain essential for maintaining medical education standards.

## Figures and Tables

**Table 1:** Overall Performance Evaluation of AI-Generated Craniofacial Anatomy Illustrations.

MEAN ± SD	Detail	Aesthetic	Usability	Cost Effectiveness
Midjourney v6.0	2.63 ± 1.24	3.53 ± 1.37	2.35 ± 1.27	2.34 ± 1.32
DALL-E 3	2.79 ± 1.15	3.49 ± 1.16	2.41 ± 1.19	2.32 ± 1.25
Stable Diffusion 2.0	1.33 ± 0.47	1.34 ± 0.65	1.17 ± 0.32	1.16 ± 0.33
Gemini Ultra	2.28 ± 1.05	2.75 ± 1.16	1.89 ± 1.03	1.85 ± 1.04
MEDIAN	Detail	Aesthetic	Usability	Cost-Effectiveness
Midjourney v6.0	3	4	2	2
DALL-E 3	3	4	2	2
Stable Diffusion 2.0	1	1	1	1
Gemini Ultra	2	3	1	1
MODE	Detail	Aesthetic	Usability	Cost-Effectiveness
Midjourney v6.0	1	1	1	4
DALL-E 3	1	1	1	1
Stable Diffusion 2.0	1	1	1	1
Gemini Ultra	1	1	2	3

ANOVA results showed statistically significant differences between the models for each evaluation criterion (Detail, Aesthetic, Usability, and Cost Effectiveness) with *p* < 0.005. Tukey’s HSD post-hoc test was used to identify where those differences occurred (see text for details).

**Table 2:** Performance of AI models in various anatomical layers.

Layer	Evaluation Criterion	Midjourney v6.0	DALL-E 3	Stable Diffusion 2.0	Gemini Ultra 1.0
Surface Anatomy	Anatomical Detail	3.78	3.65	1.84	1.28
	Aesthetic Quality	3.92	3.98	1.76	1.76
	Usability	3.45	3.45	1.55	1.38
	Cost-Effectiveness	3.49	3.51	1.52	1.29
Bones	Anatomical Detail	3.28	3.11	1.80	2.82
	Aesthetic Quality	4.27	4.04	2.30	3.12
	Usability	2.99	2.81	1.55	2.29
	Cost-Effectiveness	2.98	2.66	1.46	2.23
Muscles	Anatomical Detail	2.68	2.78	1.13	2.59
	Aesthetic Quality	3.83	3.48	1.08	3.58
	Usability	2.34	2.40	1.03	2.33
	Cost-Effectiveness	2.44	2.43	1.01	2.31
Blood Vessels	Anatomical Detail	1.88	2.78	1.13	2.22
	Aesthetic Quality	3.14	3.48	1.04	2.43
	Usability	1.62	2.40	1.01	1.72
	Cost-Effectiveness	1.55	2.43	1.01	1.62
Nerves	Anatomical Detail	1.55	2.29	1.05	1.85
	Aesthetic Quality	2.56	3.07	1.01	2.24
	Usability	1.35	1.65	1.01	1.34
	Cost-Effectiveness	1.27	1.41	1.01	1.29

**Table 3:** Agreement by reviewers in their rating is represented by Interclass Correlation Coefficients (ICC).

Level of Analysis	AI Model/Metric	ICC	95% Confidence Interval
Overall Agreement	All Models/Metrics	0.858	[0.851, 0.866]
Agreement by Model	Midjourney v6.0	0.825	[0.806, 0.843]
	DALL-E 3	0.805	[0.784, 0.825]
	Stable Diffusion 2.0	0.707	[0.675, 0.737]
	Gemini Ultra 1.0	0.776	[0.748, 0.802]
Agreement by Metric	Anatomical Detail	0.852	[0.836, 0.868]
	Aesthetic Quality	0.852	[0.835, 0.867]
	Usability	0.838	[0.820, 0.855]
	Cost-Effectiveness	0.836	[0.817, 0.853]

**Table 4:** Results of Cephalometric Analysis.

	Facial Index	Upper Facial Index	Facial Convexity Angle	Cephalic Index
Reference Image	86.22	48.40	170.96	80.00
Midjourney v6.0	86.63	47.65	173.40	84.33
DALL E-3	90.38	47.77	171.00	80.86
Stable Diffusion 2.0	93.37	52.96	176.10	75.89
Gemini Ultra 1.0	N/A	N/A	N/A	90.57

## Data Availability

The data supporting the findings of this study are available within the article and its Supplementary Materials.

## Supplementary Materials

The following supporting information can be downloaded at https://www.mdpi.com/article/10.3390/jcm14072136/s1, Table S1: Summary of intraclass correlation coefficients (ICC); Table S2: Summary of Kappa coefficients by rounding average rating score to integers (1–5).

## References

[1] Rubalcava N.S., Gadepalli S.K. (2021). From Ancient Texts to Digital Imagery: A Brief History on the Evolution of Anatomic Illustrations. Am. Surg..

[2] Chabrier R., Janke C. (2018). The comeback of hand drawing in modern life sciences. Nat. Rev. Mol. Cell Biol..

[3] Won H.S., Yang M., Kim Y.D. (2024). Necessity of professional medical illustration for increasing the value of the journal. Korean J. Pain..

[4] (2024). Becoming a Medical Illustrator: A Comprehensive Guide|Huzzle. https://www.huzzle.app/blog/becoming-a-medical-illustrator-a-comprehensive-guide.

[5] (2023). How to Become a Medical Illustrator (Skills & Steps). https://illustratorhow.com/become-medical-illustrator.

[6] (2024). Learn About Medical Illustration. https://ami.org/medical-illustration/learn-about-medical-illustration.

[7] (2023). Generative AI Has an Intellectual Property Problem. https://hbr.org/2023/04/generative-ai-has-an-intellectual-property-problem.

[8] Medical Artist Resource. http://medical-artist.org/medical-illustration-mediums.

[9] Radzi S., Chandrasekaran R., Peh Z.K., Rajalingam P., Yeong W.Y., Mogali S.R. (2022). Students’ learning experiences of three-dimensional printed models and plastinated specimens: A qualitative analysis. BMC Med. Educ..

[10] Louie P., Wilkes R. (2018). Representations of race and skin tone in medical textbook imagery. Soc. Sci. Med..

[11] Beresheim A., Zepeda D., Pharel M., Soy T., Wilson A.B., Ferrigno C. (2024). Anatomy’s missing faces: An assessment of representation gaps in atlas and textbook imagery. Anat. Sci. Educ..

[12] Chesher C. (2023). The emergence of autolography: The ‘magical’ invocation of images from text through AI. Media Int. Aust..

[13] Noel G. (2023). Evaluating AI-powered text-to-image generators for anatomical illustration: A comparative study. Anat. Sci. Educ..

[14] Totlis T., Natsis K., Filos D., Ediaroglou V., Mantzou N., Duparc F., Piagkou M. (2023). The potential role of ChatGPT and artificial intelligence in anatomy education: A conversation with ChatGPT. Surg. Radiol. Anat..

[15] Leng L. (2024). Challenge, integration, and change: ChatGPT and future anatomical education. Med. Educ. Online.

[16] Mogali S.R. (2024). Initial impressions of ChatGPT for anatomy education. Anat. Sci. Educ..

[17] Liu Z., Smith P., Lautin A., Zhou J., Yoo M., Sullivan M., Li H., Deyer L., Zhou A., Yang A. (2024). RadImageGAN–A Multi-Modal Dataset-Scale Generative AI for Medical Imaging.

[18] Ajmera P., Nischal N., Ariyaratne S., Botchu B., Bhamidipaty K.D.P., Iyengar K.P., Ajmera S.R., Jenko N., Botchu R. (2024). Validity of ChatGPT-generated musculoskeletal images. Skelet. Radiol..

[19] Goodfellow I.J., Pouget-Abadie J., Mirza M., Xu B., Warde-Farley D., Ozair S., Courville A., Bengio Y. (2014). Generative Adversarial Networks. arXiv.

[20] Ho J., Jain A., Abbeel P. (2020). Denoising Diffusion Probabilistic Models. arXiv.

[21] Ronneberger O., Fischer P., Brox T. (2015). U-Net: Convolutional Networks for Biomedical Image Segmentation.

[22] Williams M.C., Williams S.E., Newby D.E. (2023). Artificial Intelligence–based Text-to-Image Generation of Cardiac CT. Radiol. Cardiothorac. Imaging.

[23] Buzzaccarini G., Degliuomini R.S., Borin M., Fidanza A., Salmeri N., Schiraldi L., Di Summa P.G., Vercesi F., Vanni V.S., Candiani M. (2024). The Promise and Pitfalls of AI-Generated Anatomical Images: Evaluating Midjourney for Aesthetic Surgery Applications. Aesthetic Plast. Surg..

[24] Kumar A., Burr P., Young T.M. (2024). Using AI Text-to-Image Generation to Create Novel Illustrations for Medical Education: Current Limitations as Illustrated by Hypothyroidism and Horner Syndrome. JMIR Med. Educ..

[25] Kim J., Um R., Lee J., Ajilore O. (2024). Generative AI can fabricate advanced scientific visualizations: Ethical implications and strategic mitigation framework. AI Ethics.

[26] Adams L.C., Busch F., Truhn D., Makowski M.R., Aerts H., Bressem K.K. (2023). What Does DALL-E 2 Know About Radiology?. J. Med. Internet Res..

[27] Wong C. (2024). AI-generated images and video are here: How could they shape research?. Nature.

[28] (2024). Midjourney Terms of Service. https://docs.midjourney.com/docs/terms-of-service.

[29] Zhu L., Mou W., Wu K., Zhang J., Luo P. (2024). Can DALL-E 3 Reliably Generate 12-Lead ECGs and Teaching Illustrations?. Cureus.

[30] (2024). DALL·E 3. https://openai.com/index/dall-e-3.

[31] (2024). Gemini-Google DeepMind. https://deepmind.google/technologies/gemini/#introduction.

[32] (2024). Stabilityai/Stable-Diffusion-2 · Hugging Face. https://huggingface.co/stabilityai/stable-diffusion-2.

[33] Porter J.P. (2004). The average African American male face: An anthropometric analysis. Arch. Facial Plast. Surg..

[34] Moshkelgosha V., Fathinejad S., Pakizeh Z., Shamsa M., Golkari A. (2015). Photographic Facial Soft Tissue Analysis by Means of Linear and Angular Measurements in an Adolescent Persian Population. Open Dent. J..

[35] Kaya K.S., Türk B., Cankaya M., Seyhun N., Coşkun B.U. (2019). Assessment of facial analysis measurements by golden proportion. Braz. J. Otorhinolaryngol..

[36] Daniel I., Taub J.M.S.J., Jacobs J.S. (2016). Anthropometry, cephalometry, and orthognathic surgery. Plast. Surg. Key.

[37] Morosini I.d.A.C., Peron A.P.L.M., Correia K.R., Moresca R. (2012). Study of face pleasantness using facial analysis in standardized frontal photographs. Dent. Press. J. Orthod..

[38] Meneghini F., Biondi P. (2012). Clinical Facial Analysis: Elements, Principles, and Techniques.

[39] Milos Brandenberg D., González Espinoza D., Valenzuela-Fuenzalida J., Nova-Baeza P., Orellana-Donoso M. (2023). Anatomical Characteristics, Relations, and Clinical Considerations of the Facial Index and Cephalic Index in Young Chileans Aged Between 18 and 21 Years. Int. J. Morphol..

[40] Samuel S. (2024). Black Nazis? A Woman Pope? That’s Just the Start of Google’s AI Problem. Vox. https://www.vox.com/future-perfect/2024/2/28/24083814/google-gemini-ai-bias-ethics.

[41] Vaira L.A., Lechien J.R., Abbate V., Allevi F., Audino G., Beltramini G.A., Bergonzani M., Bolzoni A., Committeri U., Crimi S. (2024). Accuracy of ChatGPT-Generated Information on Head and Neck and Oromaxillofacial Surgery: A Multicenter Collaborative Analysis. Otolaryngol.-Head Neck Surg..

[42] Kwon D.Y., Wang A., Mejia M.R., Saturno M.P., Oleru O., Seyidova N., Taub P.J. (2024). Adherence of a Large Language Model to Clinical Guidelines for Craniofacial Plastic and Reconstructive Surgeries. Ann. Plast. Surg..

[43] Ismail A.F.R. (2018). Medical Animation in Educational Virtual Environments and Its Effect on Medical Reality Perception. Online J. Commun. Media–Oct..

[44] Chheang V., Sharmin S., Márquez-Hernández R., Patel M., Rajasekaran D., Caulfield G., Kiafar B., Li J., Kullu P., Barmaki R.L. (2024). Towards anatomy education with generative AI-based virtual assistants in immersive virtual reality environments. Proceedings of the 2024 IEEE International Conference on Artificial Intelligence and eXtended and Virtual Reality (AIxVR).

[45] Borji A. (2023). Qualitative Failures of Image Generation Models and Their Application in Detecting Deepfakes. arXiv.

[46] Chayka K. (2023). The Uncanny Failure of A.I.-Generated Hands. New Yorker.

[47] Ali R., Tang O.Y., Connolly I.D., Abdulrazeq H.F., Mirza F.N., Lim R.K., Johnston B.R., Groff M.W., Williamson T., Svokos K. (2024). Demographic Representation in 3 Leading Artificial Intelligence Text-to-Image Generators. JAMA Surg..

[48] Pressman S.M., Borna S., Gomez-Cabello C.A., Haider S.A., Haider C., Forte A.J. (2024). AI and Ethics: A Systematic Review of the Ethical Considerations of Large Language Model Use in Surgery Research. Healthcare.

[49] Montreal AI Ethics Institute (2022). Unstable Diffusion: Ethical Challenges and Some Ways Forward. https://montrealethics.ai/unstable-diffusion-ethical-challenges-and-some-ways-forward.

[50] Li H., Moon J.T., Purkayastha S., Celi L.A., Trivedi H., Gichoya J.W. (2023). Ethics of large language models in medicine and medical research. Lancet Digit. Health.

[51] O’Malley A., Veenhuizen M., Ahmed A. (2024). Ensuring Appropriate Representation in Artificial Intelligence–Generated Medical Imagery: Protocol for a Methodological Approach to Address Skin Tone Bias. JMIR AI.

[52] Haider S.A., Borna S., Gomez-Cabello C.A., Pressman S.M., Haider C.R., Forte A.J. (2024). The Algorithmic Divide: A Systematic Review on AI-Driven Racial Disparities in Healthcare. J. Racial Ethn. Health Disparities.

[53] Roose K. (2022). AI-Generated Art Won a Prize. Artists Aren’t Happy. The New York Times.

[54] Ma L., Han J., Wang Z., Zhang D. (2023). CephGPT-4: An Interactive Multimodal Cephalometric Measurement and Diagnostic System with Visual Large Language Model. arXiv.

[55] Google Cloud Blog (2024). A developer’s guide to Imagen 3 on Vertex AI. https://cloud.google.com/blog/products/ai-machine-learning/a-developers-guide-to-imagen-3-on-vertex-ai.

[56] Lin C.-H., Gao J., Tang L., Takikawa T., Zeng X., Huang X., Kreis K., Fidler S., Liu M.-Y., Lin T.-Y. (2022). Magic3D: High-Resolution Text-to-3D Content Creation. arXiv.

[57] Alpha3d (2023). Alpha 3d-Transform Text and 2D Images into 3D Assets with Generative AI for Free. https://www.alpha3d.io/.

[58] Liu Y., Zhang K., Li Y., Yan Z., Gao C., Chen R., Yuan Z., Huang Y., Sun H., Gao J. (2024). Sora: A Review on Background, Technology, Limitations, and Opportunities of Large Vision Models. arXiv.

[59] Pavlichenko N., Ustalov D. Best Prompts for Text-to-Image Models and How to Find Them. Proceedings of the of the 46th International ACM SIGIR Conference on Research and Development in Information Retrieval.

[60] Lee K., Liu H., Ryu M., Watkins O., Du Y., Boutilier C., Abbeel P., Ghavamzadeh M., Gu S.S. (2023). Aligning Text-to-Image Models using Human Feedback. arXiv.

[61] Goceri E. (2023). Medical image data augmentation: Techniques, comparisons and interpretations. Artif. Intell. Rev..

[62] Chen W., Hu H., Saharia C., Cohen W.W. (2022). Re-Imagen: Retrieval-Augmented Text-to-Image Generator. arXiv.

[63] Frolov S., Hinz T., Raue F., Hees J., Dengel A. (2021). Adversarial Text-to-Image Synthesis: A Review. Neural Netw..

