# Supplementary material for: A Validity Analysis of Text-to-Image Generative Artificial Intelligence Models for Craniofacial Anatomy Illustration
Source: J Clin Med. 2025 Mar 21;14(7):2136. doi: 10.3390/jcm14072136 (PMC11989924; doi:10.3390/jcm14072136)
Supplement: Supplementary file 1 [file jcm-14-02136-s001.zip › jcm-3524770-supplementary.pdf]

SUPPLEMENTARY MATERIALS:

Supplementary Table S1: Summary of intraclass correlation coefficients (ICC)

Criteria	Midjourney	DALL	SD	Gemini
ICC for D	0.548	0.378	0.479	0.265
ICC for A	0.239	0.324	0.305	0.344
ICC for U	0.552	0.492	0.412	0.289
ICC for C	0.532	0.498	0.255	0.180

Supplementary Table S2: Summary of Kappa coefficients by rounding average rating score to integers (1-5)

Kappa.name	Midjourney	DALL	SD	Gemini
Light's Kappa for D	0.222	0.133	0.262	0.115
Fleiss Kappa for D	0.203	0.108	0.242	0.061
Light's Kappa for A	0.074	0.198	0.189	0.132
Fleiss Kappa for A	0.055	0.190	0.209	0.109
Light's Kappa for U	0.177	0.140	0.273	0.129
Fleiss Kappa for U	0.163	0.124	0.319	0.069
Light's Kappa for C	0.142	0.181	0.082	0.080
Fleiss Kappa for C	0.129	0.169	0.112	0.009

Light's Kappa is the average of all possible two-raters Cohen's kappa. Fleiss Kappa measures agreement across all 4 raters
